# Editorial: Developing Medicines (Drugs) derived from the Asteraceae—an opportunity in ethnopharmacology, Volume II

**DOI:** 10.3389/fphar.2023.1285815

**Published:** 2023-09-27

**Authors:** Luis Cláudio Nascimento da Silva, Dinkar Sahal, Sujogya Kumar Panda

**Affiliations:** ^1^ Laboratory of Microbial Pathogenesis, Universidade Ceuma, São Luís, Brazil; ^2^ International Centre for Genetic Engineering and Biotechnology, New Delhi, India; ^3^ Centre of Environment Climate Change & Public Health, RUSA 2.0, Utkal University, Bhubaneswar, India; ^4^ Department of Zoology, Utkal University, Bhubaneswar, India

**Keywords:** asteraceae family, drug discovery, phytochemistry, anti-apoptosis, anti-diabetic, anti-inflammatory, anti-parasitic, wound healing property

Asteraceae family is one of the most diverse flowering plants families, with about 1911 genera and 32,913 accepted species (Panda et al., 2019). Since ancient times, people have consumed various herbs of the Asteraceae family as food and as medicine. “The 2015 Nobel Prize in Physiology or Medicine awarded for the discovery of Artemisinin and Avermectin of which the former originates from genera *Artemisia* (Asteraceae family) fundamentally changed the treatment of parasitic diseases around the globe” (Panda and Luyten, 2018). Although Asteraceae family wears the hat of tremendous diversity as well as the source of several phytochemicals, research on bioactive compounds is lacking. The focus of this Research Topic is to present six articles that illustrate some ethnopharmacological facets with respect to modern-day drug discovery including phytochemistry, pharmacological investigation, *in silico* studies of drug discovery and nutraceutical developments.

Yang et al. discuss the ethnopharmacological aspects of the dried rhizome of *Atractylodes macrocephala* Koidz, one of the most used traditional medicines in China (TCM). Also known as Baizhu, the authors have presented its traditional uses which include splenic ailments, abdominal distension, diarrhea, sputum disorders, vertigo, edema, fever, and sweating. Baizhu also aids in the cessation of vaginal bleeding during pregnancy. Phytocompounds include terpenoids, alkynes, polyacetylenes, aromatic glycosides and derivatives detected in the plant are highlighted. The authors also discussed the molecular mechanisms involved in each pharmacological property of Baizhu. Importantly, this complete review also discussed application aspects, quality control, processing, toxicity and pharmacokinetics.

Another review by Gazim et al. describes the ethnopharmacological application of *Baccharis dracunculifolia* DC, a typical species of South America that grows mainly in Argentina, Brazil, Bolivia, Paraguay and Uruguay. The first sections are structured to provide a general view of botanical aspects, geographic distribution, ethnobotanical uses (mainly as a sedative, hypotensive and bronchodilator agent, treatment of cardiovascular disorders, flu and wounds) followed by phytoconstituents (phenolic acids, diterpenes, flavonoids, triterpenes, different types of glycosides and essential oils). The compounds chlorogenic acid, caffeic acid, isochlorogenic acid A, isochlorogenic acid B, isochlorogenic acid C, artepillin C, α-amyrin, β-amyrin, pentacyclic baurenyl acetate, caffeoylquinic acids, kaempferol and luteolin reported for *B. dracunculifolia* green propolis are also presented. The authors have critically discussed the experimental results of anti-inflammatory, antimicrobial, antiulcerogenic, antioxidant, antiviral and other actions of the products derived from *B. dracunculifolia*.

Although tribal medicines make use of several plants from Asteraceae family but the active compounds are not well studied using scientific approaches. Many parts of the world have continued to remain unexplored for the indigenous knowledge of the ethnic groups. One of the best examples is a study by Woldeamanuel et al. which confirms that many traditional healers from the environs of Gullele Botanical Garden (GBG) in Addis Ababa, Ethiopia depend on the gardens for their practice of traditional medicine but there is no systematic documentation of knowledge. Approximately, 800 Ethiopian medicinal plants are being used by the indigenous tribes. Following conversations with 60 traditional healers, Woldeamanuel et al. documented that not only the majority of the plants used belong to the Asteraceae family but also from the quantitative ethnobotanical analysis, the most cited plants are from this family. Interestingly the endemic plant *E. kebericho* is endangered since it is highly exploited and steps needed for the propagation and restoration of this plant are needed at GBG. Indeed, the tribes used most commonly the leaves which are crushed and pounded as fresh preparation. Interestingly, several plants are used for the treatment of infections of the skin, respiratory system, intestine and nose besides common infections like wounds, parasites, malaria, diarrhea and diseases like tuberculosis and leprosy.

Mandal and Rahaman have presented interesting ethnobotanical research that includes ethnoveterinary medicinal knowledge (EMK) among the local people in Eastern India. Authors carry out inventorisation and consensus analysis of EMK using some popular statistical indices such as factor for informants’ consensus (Fic), use value (UV), relative frequency of citation (RFC), and fidelity level (FL) *etc.* The authors observed among the reported 92 plant families, Fabaceae has the highest reported plants followed by *Malvaceae* and *Lamiaceae* (10 species) and Asteraceae family (9 species). The Asteraceae family plants include *Blumea lacera* (Burm.f.) DC., *Echinops echinatus* Roxb., *Xanthium strumarium* L., *Centipeda minima* (L.) A.Braun and Asch., *Enydra fluctuans* DC., *Baccharoides anthelmintica* (L.) Moench, *Sphaeranthus indicus* L., *Cotula anthemoides* L. and *Eclipta prostrata* (L.) L. which is used to treat different variable health conditions such as infertility, wounds, constipation, watering of eyes, retention of the placenta and urine, rhinorrhea and fever of domestic animals (cow, goat, sheep, bull and bullock). The authors conclude two plants, *C. minima* and *C. anthemoides* reported the first time with respect to the diseases cured while the tender shoot of the plant *E. echinatus*, is a new addition in EMK of India. This article reported 306 remedies for domestic animals signifying the importance of EMK among the local population in the eastern part of India. Unfortunately, like other parts of the world and as matter of concern w.r.t. the preservation of traditional knowledge, this study also confirmed that traditional knowledge is confined to the older generation.

In a review, Mohanta et al. discussed the potential of several Asteraceae family plants for the development of pharmaceutical formulations as drug and nutraceuticals to cure diabetes. The authors observed that plants of the family Asteraceae family are known worldwide for their blood-sugar- lowering effects as well for the management of diabetes, along with scientific evidence to support such claims of the traditional healers. This review explains select potential medicinal plants of Asteraceae family as a cure for diabetes with special emphasis on ethnopharmacology to modern day drug developments. In addition, the review also highlights the role of botanical preparation as nutraceuticals with clinical evidence from this family. Considering the phytochemistry developments it describes how traditional knowledge plays a powerful role in establishing the botanicals drugs from Asteraceae family as antioxidants, hepatoprotective, vasodilators, and wound healing effects, with further action for the prevention of major diseases like cardiovascular disease (CVD), liver cirrhosis, and diabetes mellitus (DM). Notwithstanding, a few studies related to active compounds testing in animal models but pharmacokinetics/pharmacodynamics in laboratory animals and clinical trials are warranted to investigate their effects, including the mechanisms of action.

Another interesting study by Chattaraj et al. in a bid to find treatment for renal stones investigated the effect of the aqueous extract of *Enhydra fluctuans* on *in vitro* crystallization of calcium oxalate. Initially, nucleation and aggregation assays evaluated the *in vitro* inhibitory activity on calcium oxalate crystallization, followed by molecular docking to analyse the interaction of 35 metabolites with 5 proteins (ethanolamine-phosphate cytidylyltransferase, Ras GTPase-activating-like protein, UDP-glucose: glycoprotein glucosyltransferase 2, RIMS-binding protein 3A and Macrophage-capping protein) that are involved in crystal-membrane interaction, crystal growth, and stone formation. Among the tested metabolites, Baicalein-7-O-diglucoside and 4′,5,6,7-Tetrahydroxy-8-methoxy isoflavone-7-O-beta-D-galactopyranosyl-(1→3)-O-beta-D-xylopyranosyl-(1→4)-O-alpha-L-rhamnopyranoside interacted with the proteins from human renal calcium oxalate stone matrix.

Indeed, the scientific community and pharmaceutical companies have failed to provide the required attention to Asteraceae family, although a Nobel Prize has already been awarded (Panda et al., 2019). Despite the excessive scientific literature in the search for new antidiabetic drugs, more work is needed to yield potent, commercially available botanical drugs from Asteraceae family. To conclude, the present Research Topic includes interdisciplinary research work augmenting the use of traditional knowledge and fostering the scientific field of ethnopharmacology globally. This Research Topic successfully gathered comprehensive interdisciplinary information in the field of drug discovery. It is valuable to explore the beneficial effects of all these plants especially for botanical drugs reported for communicable diseases using bioassay-guided purification.

We hope that this Research Topic will further inspire scientists from different research fields to make use of the gathered traditional knowledge in the search for novel compounds and translation of promising leads into drugs towards better and affordable patient care.
